# Fermented and Aged Ginseng Sprouts (*Panax ginseng*) and Their Main Component, Compound K, Alleviate Asthma Parameters in a Mouse Model of Allergic Asthma through Suppression of Inflammation, Apoptosis, ER Stress, and Ferroptosis

**DOI:** 10.3390/antiox11102052

**Published:** 2022-10-18

**Authors:** Ji Hyeon Ryu, Min Seok Woo, Dang Long Cao, Eun-Jin Kim, Yi Yeong Jeong, Eun-Ha Koh, Kye Man Cho, Sang Soo Kang, Dawon Kang

**Affiliations:** 1 Research Institute for Convergence of Biomedical Science and Technology, Pusan National University Yangsan Hospital, Yangsan 50612, Korea; 2 Department of Physiology, Institute of Health Sciences, College of Medicine, Gyeongsang National University, Jinju 52727, Korea; 3 Department of Convergence Medical Science, Gyeongsang National University, Jinju 52727, Korea; 4 Department of Allergy and Respiratory Medicine, Gyeongsang National University and Gyeongsang National University Hospital, Jinju 52727, Korea; 5 Department of Laboratory Medicine, Institute of Health Sciences, College of Medicine, Gyeongsang National University, Jinju 52727, Korea; 6 Department of GreenBio Science, Agri-Food Bio Convergence Institute, Gyeongsang National University, Jinju 52727, Korea; 7 Department of Anatomy, Institute of Health Sciences, College of Medicine, Gyeongsang National University, Jinju 52727, Korea

**Keywords:** asthma, compound K, ferroptosis, ginseng sprout, oxidative stress

## Abstract

The association between asthma and oxidative stress remains controversial. Oxidative stress-induced ferroptosis has not been extensively studied in asthma models. This study was performed to investigate the anti-asthmatic and anti-ferroptotic effects of fermented and aged ginseng sprouts (FAGS) with enhanced antioxidant activity and its main component, compound K (CK), in a mouse model of ovalbumin (OVA)-induced allergic asthma. The experimental asthma model was sensitized and challenged with OVA. During the challenge period, two different concentrations of FAGS and CK were administered via oral gavage. Asthmatic parameters were analyzed in bronchoalveolar lavage fluid (BALF), blood, and lung tissue. CK, among the ginsenosides analyzed, was highly increased in FAGS compared with GS. Asthma parameters, such as Th2 cytokine and IgE production, mast cell activation, goblet cell hyperplasia, hyperresponsiveness, and inflammation, were dramatically increased in the OVA group. Oxidation and ferroptosis markers were increased in the OVA group. The asthma parameters and ferroptosis markers were markedly decreased in the OVA + FAGS and OVA + CK groups. These results showed that FAGS and CK alleviated asthma parameters in an allergic asthma mouse model by inhibiting inflammation and ferroptosis. Our findings suggest that FAGS and CK could be used as potential treatments for allergic asthma.

## 1. Introduction

Asthma is a chronic inflammatory respiratory disease of the airways characterized by hyperresponsiveness, inflammation, cell damage and remodeling, overexpression of T helper type 2 (Th2) cytokines, and immunoglobulin E (IgE) and mucus overproduction [1,2]. Asthma has significant negative effects on public health, with high rates of morbidity and mortality in patients affected by severe asthma. Each year, the disease claims up to 250,000 lives globally [3]. Corticosteroids are key anti-asthmatic drugs with antioxidant and anti-inflammatory effects, but increased oxidative stress has decreased the responsiveness to corticosteroids in asthma [4]. Airway inflammation is highly related to increased oxidative stress [5,6]. Recent studies have shown that oxidative stress, specifically lipid peroxidation, is related to asthma pathophysiology and severity [7,8,9]. However, the link between oxidative stress and asthma pathogenesis remains controversial because antioxidant therapy has not been clinically successful in treating asthma [10]. It is necessary not only to investigate the relationship between oxidative stress and asthma but also to find a way to prevent and treat the increase in inflammation and oxidative stress in the daily life of asthmatics.

As people’s awareness of the relationship between diet and health increases, interest in sprout vegetables is growing. Sprouts contain all the nutrients needed for growth and are known to contain more vitamins and enzymes than mature vegetables [11]. Ginseng (*Panax ginseng* Meyer) sprouts (GS) have recently started to be cultivated as a medicinal vegetable in Korea. Within 25 days of planting, ginseng can produce sprouts that are still rich in bioactive compounds, such as ginsenosides and amino acids [12,13]. Ginsenosides are highly accumulated in the shoots of 1- to 2-year-old ginseng plants [14]. The shortcomings of GS can be overcome by increasing ginsenoside content through fermentation and aging processes using microorganisms [15,16]. A recent study showed that the fermentation and aging processes increase ginsenoside F2 and compound K (CK) contents in GS 2.1-fold and 5.4-fold, respectively. In addition, the antioxidant capacity can approximately double with the increase in total phenolic and total flavonoid contents in fermented and aged ginseng sprouts (FAGS) [17]. Since the anti-inflammatory effect of ginseng is already well known, it was inferred that FAGS with increased antioxidant effect could be effective for asthma.

CK is a highly elevated component in FAGS [17]. CK, which is not found in natural ginseng, is a secondary ginsenoside biotransformed from primary ginsenosides by fermentation and intestinal microbiota [18]. CK has higher bioavailability and solubility than the parent ginsenosides [18]. CK has been discovered in human tissues or blood after the oral consumption of protopanaxadiol (PPD)-type ginsenoside and red ginseng extract [19,20]. The reason why antioxidants are not clinically successful in asthma treatment is thought to be their ineffective in vivo metabolism and stability. CK and FAGS could be agents that can compensate for the shortcomings of other antioxidants. Many types of ginseng, but not FAGS, have been shown to be effective in asthma models, regulating inflammatory, apoptotic, and autophagy mechanisms [18,21,22]. The effect of CK has not been adequately studied in asthma models [23]. On the other hand, CK has been shown to have a protective effect on various diseases by inhibiting apoptosis and ER stress as well as inflammation [18]. Apoptosis and ER stress are associated with the onset of asthma [24,25], and ER stress regulates airway inflammation, apoptosis, and airway remodeling [26]. However, there are still limitations to the detailed mechanism analysis applicable to clinics.

A recent study showed that a high iron level in lung cells is associated with asthma exacerbations [27]. Elevated iron levels induce key asthmatic characteristics, such as airway hyperresponsiveness, fibrosis, and Th2 inflammatory responses [27]. This study motivated us to determine whether ferroptosis, a mechanism associated with oxidative stress, is involved in the pathogenesis of asthma. The mechanism of ferroptosis has not been extensively studied in asthma models, and its inhibitors are not well known. This study was performed to determine whether FAGS and CK modulate ferroptosis signaling and exert anti-asthmatic effects in a mouse model of OVA-induced allergic asthma.

## 2. Materials and Methods

### 2.1. Preparation of Fermented and Aged Ginseng Sprouts Extract

Ginseng sprouts (GS) produced using the soil-substrate cultivation method were provided by Dream Farm Cop. (Jinju, Korea). These ginseng seedlings (2 years old) were cultivated in a plant factory (Smart Farm Cube; Dream Farm Cop.) under artificial white, red, and blue LED lights, temperatures of 19 ± 2 °C, relative humidity of 60 ± 5%, and 400 µmol/mol CO_2_. They were cultivated for 30 days in a plant factory that automatically controlled the conditions. The GS sample was prepared by washing them three times in water and cutting them into the length of approximately 1 cm. Washed GS were steamed at 95 ± 2 °C for 60 min and then aged at 75 ± 2 °C for 72 h in an aging chamber. The steaming and aging processes were repeated three times. Aged GS (AGS) were put in a dry oven set at 55 ± 2 °C for 72 h to remove the water. AGS (200 g) were added separately to 400 mL of tap water with 2% sucrose in a 500 mL stainless steel container. The mixture was first sterilized at 121 °C for 30 min, and then cooled to 35 ± 2 °C. The precultured *Lactobaciius brevis* BMK184 and *Lactobacillus plantarum* P1201 strains were inoculated at cell densities of 8.2 log cfu/mL and 8.0 log cfu/mL, respectively [17]. The AGS substrate was fermented at 35 ± 1 °C for 120 h with the two lactic acid bacteria (*L. brevis* BMK184 and *L. plantarum* P1201) precultured in a lactobacilli MRS broth or agar (MRSB or MRSA; Difco, Becton Dickinson Co., Sparks, MD, USA) [16]. The fermented AGS (FAGS) samples were freeze-dried and kept at −70 ± 1 °C until analysis.

### 2.2. Analysis of Ginsenoside Compounds

The quantification of ginsenoside derivatives was carried out using a high performance liquid chromatography (HPLC) 1260 system (Agilent Technologies Inc., Waldbronn, Germany) with previously described methods [16]. This system included an Aligent 1260 diode-array detector (DAD), a quaternary pump, an autosampler, TSK-ODS100Z (ginsenosides; Tosoh Corp., Tokyo, Japan), and an X-Bridge C18-RP column (flavonols and phenolic acids; Waters Corp., Milford, MA, USA). For the ginsenoside analyses, 50 mL of 50% methanol (MeOH) was added to 1 g of GS and FAGS powders, and the mixtures were extracted at 70 ± 2 °C for 1 h. The supernatant was recovered from the extracts by filtering them twice using a 0.45 µm membrane filter. Each extract supernatant was concentrated and dried utilizing a rotary evaporator. Finally, 2 mL of acetonitrile: H_2_O (8:2, *v*/*v*) was added to the dried extracts to dissolve them. The dissolved samples were filtered through a 0.45 µm membrane filter for the HPLC analysis. The ginsenoside standards were bought from KOC Biotech Co., Ltd. (Daejeon, Korea) and Sigma-Aldrich Chemical Co. (St. Louis, MO, USA).

### 2.3. Preparation of 50% EtOH Extract Concentrates for Animal Experiments

The mixtures (20 g of GS or FAGS powder and 400 mL of 50% EtOH) were extracted at 40 ± 2 °C for 5 h. The supernatant was obtained from the extract by filtering it twice using a 0.45 µm membrane filter. Each extract supernatant was concentrated to approximately 18° Brix and filtered to create the extract concentrates using a rotary evaporator.

### 2.4. Animal Care

Six-week-old female C57BL/6 mice were purchased from the Koatech animal breeding center (Pyeongtaek, Korea). The mice were kept in a specified pathogen-free animal facility under a 12-h light/dark cycle with unrestricted access to food and water. On the day of operation and every week, mice’s body weights were measured. The Gyeongsang National University Animal Care and Use Committee’s guidelines were followed during animal experiments (GNU-210308).

### 2.5. Induction of Allergic Asthma and Treatment

All mice were randomly divided into five groups (*n* = 5~10) as follows: sham control (CTL, *n* = 10), OVA (*n* = 10), OVA + low dose of FAGS (LFAGS; 300 mg/kg/day, *n* = 5), OVA + high dose of FAGS (HFAGS; 600 mg/kg/day, *n* = 10), and OVA + CK (50 μM/kg/day, *n* = 5). The protocol for the induction and treatment of allergic asthma was modified based on a previous study [28]. On days 0, 7, and 14, 100 µg of OVA (Hyglos GmbH, Regensburg, Germany) dissolved in 200 μL of phosphate-buffered saline (PBS) with 2 mg of aluminum hydroxide (alum; Invivo Gen, SanDiego, CA, USA) was intraperitoneally injected into each mouse for sensitization. Mice were intranasally challenged with 50 µg of OVA in PBS three times a week for 3 weeks after being given isoflurane (Hana Pharm Co., Ltd., Hwaseong, Korea; 2% induction and 1.5% maintenance, in 80% N_2_O and 20% O_2_) to anesthetize them. At the time of OVA injection, CTL mice were sensitized and challenged with PBS. FAGS and CK were orally administrated daily from day 21 to day 40 of OVA challenge. At the same time, FAGS was administered, distilled water was given via oral gavage to CTL mice. The methacholine (MCh) test was performed 24 h after the last challenge was completed. All mice were sacrificed for analyses two days following the last OVA challenge. The spleen and thymus were isolated and weighed on the day of operation.

### 2.6. MCh Test

The increase in pulmonary resistance upon challenge with aerosolized MCh in awake mice was assessed to determine the degree of airway hyperresponsiveness using a whole-body plethysmograph (OCP 3000; Allmedicus, Gyeonggi, Korea) as previously described [28]. Briefly, mice were exposed to 25 and 50 mg/mL of MCh (Sigma) for 10 min using a nebulizer (HARVARD73-1963; Harvard Apparatus, MA, USA). After each nebulization, mice were immediately put back in their chambers, and the measurement was taken 150 s later. Enhanced pause (Penh) was determined based on the mean pressure produced in the plethysmography chamber.

### 2.7. Collection of Bronchoalveolar Lavage Fluid (BALF) and Differential Cell Count

BALF collection and differential cell counts were performed as previously described [28]. Mice were sacrificed with avertin tribromoethanol (Sigma) at a lethal dosage to collect BALF; then, the lungs were lavaged with 1 mL of ice-cold PBS using a tracheostomy tube. The total number of BAL cells was counted after BALF was centrifuged at 300*× g* for 10 min at 4 °C. For further cytokine analyses, BALF samples were frozen at −80 °C. Differential cells were allowed to adhere to a slide via cytocentrifugation (Cytospin; Thermo Shandon, Pittsburgh, PA, USA) and stained with Diff-Quick (Sysmex International Reagents, Kobe, Japan). Neutrophils, eosinophils, lymphocytes, or macrophages were sorted when counting more than 300 cells.

### 2.8. Measurement of Total and OVA-Specific Immunoglobulin E (IgE) in Plasma

Total and OVA-specific IgE levels in plasma were determined using a Mouse IgE ELISA kit (Bethyl Laboratories, Montgomery, TX, USA) and an Anti-Ovalbumin IgE ELISA kit (Cayman Chemical, Ann Arbor, MI, USA), respectively, following the manufacturer’s instructions and the procedures described in a previous study [29]. The IgE level of each sample in the experimental group was measured using absorbance, and absorbance was read at 450 nm with a microplate reader (Tecan; Infinite™ M200 PRO; Männedorf, Switzerland).

### 2.9. Immunoassay for T helper Type 2 (Th2) Cytokines, Histamine, and Tryptase in BALF

The concentrations of Th2 cytokines (IL-4, IL-5, and IL-13) and histamine were quantified using ELISA kits (Th2 cytokines, R&D System, Minneapolis, MN, USA; histamine, Enzo Life Sciences, Ann Arbor, MI, USA) in accordance with the manufacturer’s instructions and a modified experimental procedure from a previous study [29]. For the measurement of mast cell tryptase concentration using an ELISA kit (Cusabio Biotech, Wuhan, China), 100 µL of BALF was added to a 96-well plate and incubated at 37 °C for 2 h; then, the sample was taken out. Next, 100 µL of biotin antibody was added, incubated at 37 °C for 1 h, and washed thrice. Then, 100 µL of HRP-avidin was added, incubated at 37 °C for 1 h, and washed five times. Finally, 90 µL of TMB substrate solution was added, and the sample was incubated at 37 °C for 20 min. By adding 50 µL of stop solution, the reaction was quenched. Using a microplate reader (Tecan), the absorbance of Th2 cytokines, histamine, and mast cell tryptase on the plates was measured at 450 nm.

### 2.10. Lung Histology

Lung inflammation and mucus production were analyzed using hematoxylin and eosin (H&E; Sigma) and periodic acid–Schiff (PAS; Millipore, Billerica, MA, USA) staining, respectively. Staining was performed according to a previous protocol [29]. Collagen deposition was analyzed using Masson’s trichrome (MT; Polysciences, Warrington, PA, USA). Deparaffinized sections were subjected to a fixative at 60 °C for 1 h, Weigert’s iron hematoxylin for 10 min, Biebrich Scarlet Acid Fuchsin for 5 min, and Phosphotungstic/phosphomolybdic acid for 10 min; then, they were washed in acetic acid. Five sections from each sample were examined after being photographed using a virtual microscope (Axio Scan.Z1; Carl Zeiss, Jena, Germany). Lung inflammation severity in H&E sections was rated on the following scale: no cell (0); a few cells (1); a ring of cells, 1 cell layer deep (2); a ring of cells, 2–4 cell layers deep (3); and a ring of cells, >4 cell layers deep (4) [30]. The number of mucin-positive goblet cells (PAS sections) was rated using the following scale: <5% PAS-positive cells (0); 5–25% (1); 25–50% (2); 50–75% (3); and >75% (4) [31]. The intensity of collagen deposition in MT sections was rated on the following scale: no collagen deposition (0); a thin layer of collagen (1); a cluster of collagen (2); and a thick layer of collagen (3) [32].

### 2.11. Prussian Blue Staining for Iron in Lung Tissue

Paraffin sections of lung tissue were deparaffinized and hydrated with distilled water. Equal parts of potassium ferrocyanide (10%) and hydrochloric acid (20%) were combined and produced before use. The lung tissue slides were submerged in this solution for 20 min and then rinsed three times in distilled water. The slides were counterstained with a nuclear fast red solution for 5 min and cleaned twice in distilled water. Finally, the slides were mounted using a resinous mounting medium after being dehydrated.

### 2.12. Immunohistochemistry in Lung Tissue

Paraffin sections of lung tissue were deparaffinized and hydrated with distilled water. The deparaffinized lung tissue slices were permeabilized with 0.2% Triton X-100 at room temperature for 10 min. The tissue slices were washed three times in PBS and then incubated at room temperature for 60 min with blocking buffer (10% normal goat serum in 0.1 M PBS). The slices were then exposed to rabbit polyclonal anti-CD68 primary antibody (1:100 dilutions; Santa Cruz Biotechnology, Dallas, TX, USA) for overnight incubation at 4 °C. The slices were treated with Alexa Fluor 488-conjugated goat anti-rabbit IgG secondary antibody (1:300 dilutions; Invitrogen, Waltham, MA, USA) for 60 min in the dark following three washes in PBS. Finally, the sections were washed three times in PBS and stained with Hoechst 33342 (0.1 µg/mL; Sigma) for nucleus staining. Using a confocal laser scanning microscope (Olympus, Tokyo, Japan), the stained slices were observed after being wet-mounted with a Permount mounting medium (Fisher Chemical, Geel, Belgium).

### 2.13. Measurement of Free Radical Activity and Calcium Concentration in Lung Tissue

The free radical activity was assessed in lung tissue lysates using an Oxiselect^TM^ In Vitro ROS/RNS assay kit (Cell Biolabs, San Diego, CA, USA) as previously described [33]. Calcium concentration was measured using a Calcium Detection Assay kit (Abcam) as previously described [34].

### 2.14. Measurement of Malondialdehyde (MDA) Concentration in Lung Tissue

According to the manufacturer’s protocol, MDA concentration was assessed with an OxiSelect™ TBARS assay kit (STA-330; Cell Biolabs). Butylated hydroxytoluene (BHT; 1×) was added to each sample (50 mg/mL in PBS) to halt further oxidation. Tissue lysates were centrifuged at 10,000× *g* for 10 min at 4 °C. The resulting supernatant served as an assay sample. MDA standards in the 125 to 0 μM were produced via serial dilution in distilled water. Samples or standards (100 μL) were placed in microcentrifuge tubes, and sodium dodecyl sulfate (SDS) lysis solution (100 μL) was added, mixed thoroughly, and then incubated for 5 min at room temperature. The tubes were then filled with thiobarbituric acid (TBA; 250 μL), well mixed, incubated at 95 °C for 45 min, and cooled to room temperature via incubation in ice for 5 min. Samples were centrifuged at 3000 rpm at room temperature for 15 min, and the resulting supernatants were collected in microcentrifuge tubes; then, 200 μL of standards and samples were placed in each well of a 96-well plate. The absorbance of samples and standards was read at 532 nm using a VERSA max microplate reader (Molecular Devices, San Jose, CA, USA), and the concentration was calculated from the MDA standard curve.

### 2.15. Terminal Deoxynucleotidyl Transferase dUTP Nick end Labeling (TUNEL) Assay

Using a DeadEnd Fluorometric TUNEL system (Promega, Madison, WI, USA), the apoptotic signal in the lung tissue slice was evaluated in accordance with the manufacturer’s instructions. TUNEL staining was performed as previously described [35]. TUNEL-positive cells were counted using a fluorescent microscope (Leica, Wetzlar, Germany).

### 2.16. Western Blot Analysis

Western blot analysis was carried out as previously described [35]. Total proteins were extracted from lung tissue using the radioimmunoprecipitation assay (RIPA) buffer (Thermo Fisher Scientific, Rockford, IL, USA), which contained 1× protease inhibitor cocktail (Roche Diagnostics, IN, USA). Tissue lysates incubated on ice for 30 min were centrifuged at 16,600× *g* (13,000 rpm; Eppendorf, Hamburg, Germany) for 20 min at 4 °C. For nuclear and cytoplasmic fractions, the lung tissue lysates homogenized in a hypotonic lysis buffer were centrifuged with 142× *g* for 5 min at 4 °C. The resulting pellets and supernatants were used for the isolation of nuclear and cytoplasmic fractions, respectively. The detection of NF-κB activation was performed with previously described methods [34]. The cytosolic and nuclear fractions were verified to be free of contaminating nucleus and cytosol by immunoblotting for the cytoplasmic marker β-actin and the nuclear marker lamin, respectively. The amount of protein in the tissue lysates was measured using a Pierce bicinchoninic acid (BCA) protein assay kit (Thermo Fisher Scientific, Rockford, IL, USA). Equal quantities of proteins mixed with 5× SDS sample buffer were separated on mini gels (Novex™ WedgeWell™ 4 to 12% Tris-glycine; Thermo Fisher Scientific) by electrophoresis for 25 min at 200 V, and the gel was blotted onto a polyvinylidene difluoride (PVDF; Millipore, Bedford, MA, USA) membrane for 60 min at 100 V using the wet-transfer method. Membranes were first blocked at room temperature for 60 min with 5% (*w*/*v*) fat-free dried milk in a solution of 20 mM Tris HCl (pH 8.0), 150 mM NaCl, and 0.1% Tween-20 (TBST). The membranes were then incubated with anti-caspase-3 (1:1000; Cell Signaling, Danvers, MA, USA), anti-B-cell lymphoma protein 2 (Bcl-2; 1:200 dilution; Santa Cruz Biotechnology), anti-Bcl-2-associated X (Bax; 1:200 dilution; Santa Cruz Biotechnology), anti-protein kinase R-like ER kinase (PERK; 1:200 dilution; Santa Cruz Biotechnology), anti-p-PERK (1:200 dilution; Santa Cruz Biotechnology), anti-eukaryotic initiation factor 2α (elF2α; 1:1000 dilution; Cell Signaling), anti-p-elF2α (1:1000 dilution; Cell Signaling), anti-activating transcription factor (ATF4; 1:200 dilution; Santa Cruz Biotechnology), anti-C/EBP homologous protein (CHOP; 1:200 dilution; Santa Cruz Biotechnology), anti-solute carrier family 7 member 11 (SLC7A11; 1:1000 dilution; Arigo Biolaboratories Corp, Hsinchu City, Taiwan), anti-glutathione peroxidase 4 (GPX4; 1:1000 dilution; Arigo Biolaboratories Corp), anti-4-Hydroxynonenal (4-HNE; 1:1000 dilution; Arigo Biolaboratories Corp), anti-NF-κB p65 (1:1000 dilution; Cell Signaling), anti-lamin B1 (1:1000 dilution; Cell signaling), and anti-β-actin (1:5000 dilution; Sigma) at 4 °C overnight. Primary antibody incubation was followed by incubation with a secondary anti-rabbit, anti-mouse, or anti-goat antibody at 1:5000 dilution (Assay Designs, Ann Arbor, MI, USA). Immunopositivity bands were developed by enhanced chemiluminescence (Thermo Fisher Scientific) and visualized with an iBright^TM^ CL1500 imaging system (Thermo Scientific Fisher/Life Technologies Holdings Pte Ltd., Singapore). The β-actin was used as a loading control to calculate the relative protein level.

### 2.17. Statistics

Data were represented as mean ± standard error (SE) except for the analysis of ginsenoside compounds. The data obtained from the analysis of ginsenoside compounds were expressed as mean ± standard deviation (SD). After the normality test, the one-way ANOVA/Bonferroni test or the Kruskal Wallis/Mann Whitney test was selected to analyze group differences (OriginPro2020; OriginLab Corp, Northampton, MA, USA). The statistical significance criterion was set at a value of *p* < 0.05.

## 3. Results

### 3.1. Contents of Ginsenoside Derivatives of GS and FAGS

**Figure 1:**
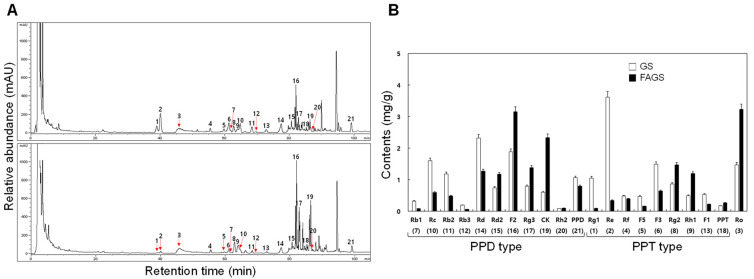
Typical HPLC chromatograms of GS and FAGS. (**A**) Relative ginsenoside contents of 50% ethanol-extract concentrates of GS and FAGS. Peaks are numbered. Arrows indicate less visible ginsenoside peaks. Numbers above the peaks indicating ginsenoside names are described in (**B**). (**B**) Comparison of ginsenoside contents in GS and FAGS. Peak numbers in parentheses indicate ginsenoside names, which are displayed below the bar graph. All values are presented as mean ± SD of pentaplicate determinations. GS and FAGS indicate ginseng sprouts and fermented and aged ginseng sprouts, respectively. PPT and PPD indicate protopanaxatriol and protopanaxadiol, respectively.

A total of 21 ginsenoside peaks were detected in the HPLC chromatogram of GS and FAGS samples (Figure 1A). As shown in Figure 1B, the major ginsenosides of GS samples were as follows, in order: Re (3.61 mg/g), Rd (2.32 mg/g), F2 (1.88 mg/g), Rc (1.60 mg/g), F3 (1.49 mg/g), Ro (1.47 mg/g), Rb2 (1.18 mg/g), protopanaxadiol (PPD, 1.06 mg/g), and Rg1 (1.05 mg/g). The major ginsenosides of FAGS samples were as follows, in order: Ro (3.23 mg/g), F2 (3.15 mg/g), CK (2.33 mg/g), Rg2 (1.47 mg/g), Rg3 (1.38 mg/g), Rd (1.27 mg/g), Rh1 (1.19 mg/g), and Rd2 (1.17 mg/g). F2 (1.88→3.15 mg/g), Rg3 (0.79→1.38 mg/g), and Rg2 (0.86→1.47 mg/g) increased approximately 1.7-fold in FAGS compared to GS. The Ro (1.47→3.23 mg/g) and Rh1 (0.49→1.19 mg/g) increased 2.2-fold and 2.4-fold, respectively, in FAGS. CK increased by 3.9-fold (0.60→2.33 mg/g) in FAGS compared with GS and was the one that was increased the most among the ginsenosides that varied (Figure 1B). Animal experiments were conducted using FAGS and CK.

### 3.2. FAGS and CK Reduced OVA-Induced Airway Hyperresponsiveness and IgE Production

**Figure 2:**
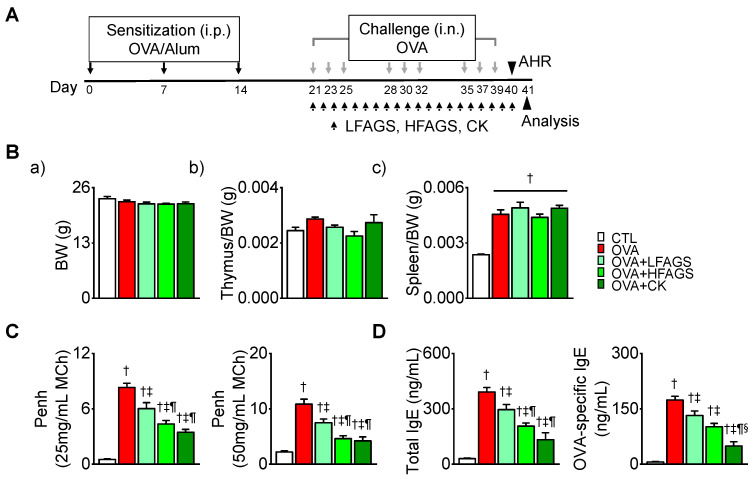
Inhibitory effects of FAGS and CK on OVA-induced airway hyperresponsiveness and IgE production. (**A**) Production of OVA-induced airway allergic asthma mouse model. Time representation of asthma model and pharmacological intervention. The i.p. and i.n. represent intraperitoneal injection and intranasal injection, respectively. AHR represents airway hyperresponsiveness. (**B**) No effects of FAGS and CK on OVA-induced changes in spleen or thymus weight. (**a**) Total body weight. (**b**) Thymus weight/body weight ratio. (**c**) Spleen weight/body weight ratio. (**C**) Assessment of airway hyperresponsiveness in OVA-challenged mice. Airway resistance to direct stimulation (Penh) was measured using MCh at 25 and 50 mg/mL. (**D**) Inhibitory effects of FAGS and CK on total and OVA-specific IgE levels in plasma. Data are shown as mean ± SE (CTL, *n* = 10; OVA, *n* = 10; OVA + LFAGS, *n* = 5; OVA + HFAGS, *n* = 10; OVA + CK, *n* = 5). ^†^ *p* < 0.05 compared with CTL. ^‡^ *p* < 0.05 compared with OVA. ^¶^ *p* < 0.05 compared with LFAGS. ^§^ *p* < 0.05 compared with HFAGS.

An allergic asthma model was produced by OVA exposure. During the challenge with OVA, two different concentrations of FAGS (LFAGS at 300 mg/kg/day and HFAGS at 600 mg/kg/day) and CK (50 μM/kg/day) were orally administered (Figure 2A). The body weight (BW) and the weights of immune organs spleen and thymus were measured on the day of surgery and compared between the experimental groups. BW and thymus weight did not change in the experimental groups (Figure 2(Ba,Bb)). The spleen’s weight was significantly increased in the OVA administered (OVA) group (*p* < 0.05). FAGS and CK did not affect the OVA-induced increase in spleen weight (Figure 2(Bc)). As shown in Figure 2C, the MCh challenge (25 mg/mL and 50 mg/mL) caused a dose-dependent increase in enhanced pause (Penh) in the OVA group. Penh was increased in the OVA group and was decreased in the FAGS and CK groups (Figure 2C). Total and OVA-specific IgE levels in plasma were significantly decreased in the FAGS and CK groups (*p* < 0.05; Figure 2D). In terms of the changes in Penh and IgE levels, CK showed an inhibitory effect that was more similar to that of HFAGS than that of LFAGS (Figure 2C,D).

### 3.3. FAGS and CK Alleviated OVA-Induced Airway Inflammation in Bronchoalveolar Lavage Fluid

**Figure 3:**
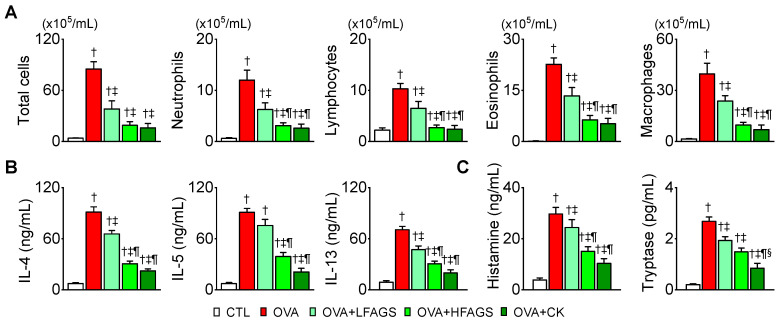
Inhibitory effects of FAGS and CK on OVA-induced increase in inflammatory cells, Th2 cytokines, histamine, and tryptase in BALF. (**A**) Total and differential inflammatory cell count in BALF. Differential cell counts were assessed using Diff-Quick staining. (**B)** Measurement of Th2 cytokines (IL-4, IL-5, and IL-13) in BALF. (**C**) Measurement of histamine and tryptase secreted from mast cells. Data are shown as mean ± SE (CTL, *n* = 10; OVA, *n* = 10; OVA + LFAGS, *n* = 5; OVA + HFAGS, *n* = 10; OVA + CK, *n* = 5). ^†^ *p* < 0.05 compared with CTL. ^‡^ *p* < 0.05 compared with OVA. ^¶^ *p* < 0.05 compared with LFAGS. ^§^ *p* < 0.05 compared with HFAGS.

The OVA group showed a significant increase in the number of inflammatory cells in BALF (*p* < 0.05). The numbers of total inflammatory cells, including neutrophils, lymphocytes, eosinophils, and macrophages, were significantly decreased in FAGS- and CK-administered groups compared with the OVA group (*p* < 0.05). Compared with the LFAGS group, the HFAGS and CK groups showed a significant decrease in the number of inflammatory cells (*p* < 0.05; Figure 3A). Their effect was approximately 2-fold higher than the LFAGS group. The inhibitory effect of HFAGS on the number of inflammatory cells was similar to that of CK (Figure 3A). The concentration of Th2 cytokines (IL-4, IL-5, and IL-13) in BALF was significantly elevated in the OVA group compared with the control (CTL) group (*p* < 0.05; Figure 3B). The OVA-induced increase in Th2 cytokine concentration was decreased in the FAGS and CK groups. The HFAGS group showed a higher inhibitory effect on IL-4, IL-5, and IL-13 concentrations than the LFAGS group. The CK group showed the highest effect on the reduction in IL-5 concentration (Figure 3B). The levels of histamine and tryptase, which are secreted from mast cells activated by Th2 cytokines, in BALF were significantly high in the OVA group (*p* < 0.05; Figure 3C). The OVA-induced increases in histamine and tryptase were markedly decreased in the FAGS and CK groups. In terms of histamine concentration, the HFAGS and CK groups showed higher inhibitory effects than LFAGS. In the OVA + CK group, tryptase concentration was significantly decreased by approximately 2-fold compared with OVA + LFAGS and OVA + HFAGS (Figure 3C).

### 3.4. FAGS and CK Reduced OVA-Induced Airway Inflammation in Lung Tissues

**Figure 4:**
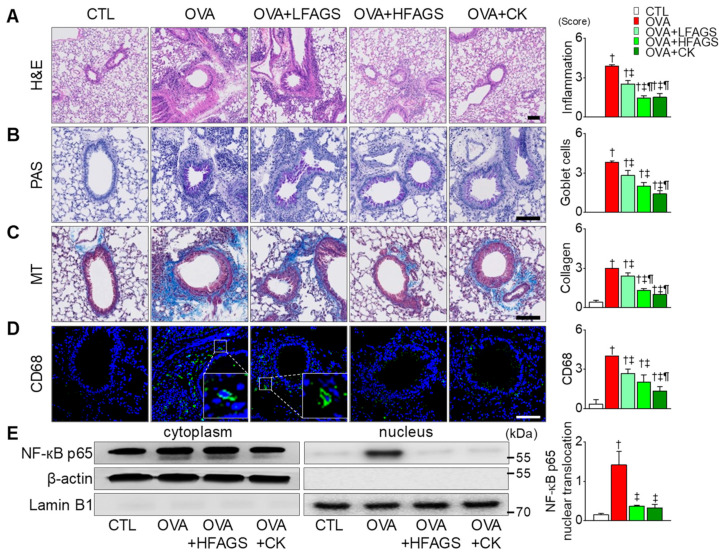
Inhibitory effects of FAGS and CK on OVA-induced allergic airway inflammation in lung tissue. (**A**) Representative images of H&E staining showing infiltration of inflammatory cells. (**B**) PAS staining performed to detect mucus-secreting goblet cells (PAS staining-positive cells). (**C**) Masson’s trichrome (MT) stains showing collagen-fiber deposition (blue). (**D**) CD68-positive cells (green). (**E**) Detection of NF-κB p65 in cytoplasmic and nuclear fractions. Bar graphs show summarized scores of inflammation (**A**), goblet cells (**B**), collagen-fiber deposition (**C**), macrophage accumulation (**D**), and NF-κB p65 nuclear translocation (**E**) in each experimental group. Scale bars, 100 μm. Data are shown as mean ± SE (each group *n* = 5). ^†^ *p* < 0.05 compared with CTL. ^‡^ *p* < 0.05 compared with OVA. ^¶^ *p* < 0.05 compared with LFAGS.

Lung tissues isolated from the OVA group showed marked infiltration of inflammatory cells into the peribronchial area compared with the CTL group. The OVA-induced infiltration of inflammatory cells was significantly decreased in the FAGS and CK groups (*p* < 0.05; Figure 4A). Goblet cells that secreted mucus were detected with PAS staining. The OVA group showed significant goblet cell hyperplasia compared with the CTL group (*p* < 0.05; Figure 4B). The number of goblet cells was significantly decreased in the OVA + FAGS and OVA + CK groups (*p* < 0.05). Masson’s trichrome (MT) staining showed accumulated collagen in the peribronchial area and in sites surrounding vascular vessels in the OVA group. In the group of FAGS or CK combined with OVA, the number of collagen fibers was decreased (Figure 4C). Macrophage infiltration into the peribronchial area was higher in the OVA group than that in the CTL group (Figure 4D). In the OVA + FAGS and OVA + CK groups, macrophage infiltration was significantly decreased (*p* < 0.05). CK effect was higher than FAGS effect in the number of goblet cells and macrophage infiltration (Figure 4B,D).

### 3.5. FAGS and CK Attenuated OVA-Induced Apoptosis, ER Stress, and Ferroptotic Signals in Lung Tissue

**Figure 5:**
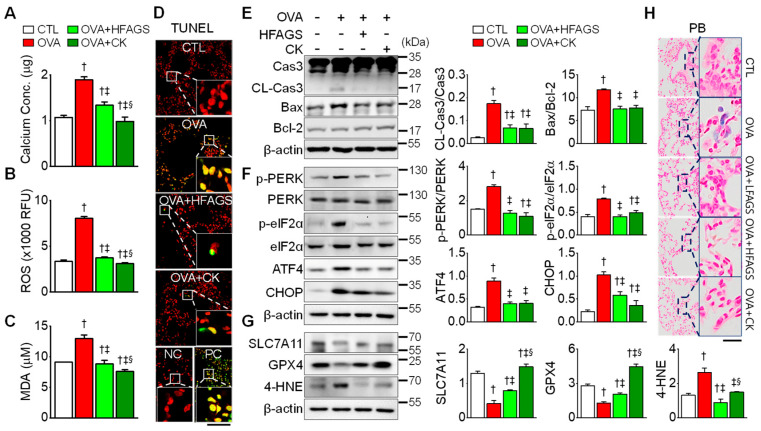
Inhibitory effects of FAGS and CK on apoptotic, ER stress, and ferroptotic signals in lung tissue obtained from OVA-exposed mice. (**A**) Calcium concentration. (**B**) ROS level. RFU represents relative fluorescence units. (**C**) MDA level. (**D**) TUNEL-positive cells. Counterstaining was performed via incubation with 5 μg/mL propidium iodide (red). Scale bar, 50 μm. NC and PC represent negative control and positive control, respectively. NC, control treated with label solution without terminal transferase; PC, control treated with DNase I. (**E**) Apoptotic signals (cleavage of caspase 3 and increase in Bax/Bcl-2 ratio). Cas3 and CL-Cas3 represent caspase 3 and cleaved caspase 3, respectively. (**F**) ER stress markers. (**G**) Ferroptotic markers. (**H**) Perls Prussian blue staining showing iron accumulation. Data are shown as mean ± SE (each group, *n* = 5). ^†^ *p* < 0.05 compared with CTL. ^‡^ *p* < 0.05 compared with OVA. ^§^ *p* < 0.05 compared with HFAGS.

Oxidative stress can lead to inflammation and cell death [36]. In the OVA group, calcium concentration in lung tissue was high compared with the CTL group (Figure 5A). The calcium concentration was significantly decreased by 30% and 48% in the OVA + HFAGS and OVA + CK groups, respectively (*p* < 0.05). The amount of reactive oxygen species (ROS) was higher in the OVA group than in the CTL group (Figure 5B). In addition, the concentration of MDA, a marker of oxidative stress, in particular, a lipid peroxidation marker, was high in the OVA group compared with the CTL group (Figure 5C). The ROS and MDA contents were reduced in the OVA + HFAGS and OVA + CK groups by approximately 60% and 40%, respectively, compared with OVA group. The number of TUNEL-positive cells was increased in the OVA group and significantly decreased in the OVA + HFAGS and OVA + CK groups (Figure 5D; *p* < 0.05). The cleavage of caspase 3 (CL-Cas 3) and the Bax/Bcl-2 ratio were increased in the OVA group (Figure 5E). The apoptotic markers were significantly decreased in the OVA + HFAGS and OVA + CK groups (*p* < 0.05).

ER stress-related proteins (PERK, eIF2α, ATF4, and CHOP) were upregulated in the OVA group. The upregulated ER stress markers were markedly decreased in the OVA + HFAGS and OVA + CK groups (Figure 5F). It is known that ferroptosis is characterized by an increase in lipid peroxide due to the disruption of glutathione-dependent antioxidant defenses [37], that ferrototic agents activate the PERK–eIF2α–ATF4–CHOP pathway [37], and that the suppression of SLC7A11 (an essential transporter of cysteine) and GPX4 (main regulator in the ferroptotic process) is linked to ferroptosis induction, while 4-HNE (lipid peroxidation product) upregulation is related to lipid peroxidation during the ferroptotic process. In our study, the SLC7A11 and GPX4 expression levels were decreased in the OVA group, while the 4-HNE expression level was increased. The OVA-induced changes in the ferroptosis markers were significantly restored in the OVA + HFAGS and OVA + CK groups (*p* < 0.05; Figure 5G). In addition, iron-positive cells were detected in higher numbers in the OVA group than in the CTL group. The number of iron-positive cells increased in the OVA group was decreased in the OVA + HFAGS and OVA + CK groups (Figure 5H).

## 4. Discussion

To the best of our knowledge, this study is the first to report that FAGS and its main component, CK, exert anti-asthmatic effects in mice exposed to OVA for 39 days through inhibiting inflammation, oxidative stress, ferroptosis, and ER stress. Various asthmatic airway parameters were investigated here, i.e., Th2 cytokine (IL-4, IL-5, and IL-13) and IgE production, histamine and tryptase secretion (mast cell activation), goblet cell hyperplasia, mucus hypersecretion, hyperresponsiveness, inflammation (increase in numbers of eosinophils, neutrophils, and macrophages), and smooth muscle spasms (increase in collagen level and Ca^2+^ concentration). In addition, ER stress (increases in Ca^2+^ concentration, PERK and eIF2α activation, and ATF4 and CHOP levels) and ferroptosis (production of ROS and MDA, downregulation of SLC7A11 and GPX4, and upregulation of 4-HNE) markers were detected in the OVA group. Apoptotic signals (cleavage of caspase 3 and increase in Bax/Bcl2 ratio and in the number of TUNEL-positive cells) were also abundant in the OVA group. These asthmatic parameters were markedly decreased in the OVA and FAGS or CK combined group. This study deepened our understanding of the close relationship between asthma pathogenesis and oxidative stress, which has not been clearly elucidated so far, by adding ER-stress and ferroptosis mechanisms to the known characteristics of asthma pathogenesis. It is likely that crosstalk among inflammation, apoptosis, ER stress, and ferroptosis signals occurs in the OVA-exposed mouse model. Here, we propose FAGS and CK as ferroptosis inhibitors as well as therapeutic agents for diseases caused by inflammation and oxidative stress, such as asthma.

Ferroptosis, a form of programmed cell death, results from the excessive accumulation of iron-dependent ROS and lipid peroxides [7]. In asthma, high levels of iron cause airway inflammation and hyperoxidative state, leading to ferroptosis [7,8]. Activated macrophages and high levels of pro-inflammatory mediators are detected in ferroptosis-affected tissues. Ferroptosis begins and progresses under intracellular iron accumulation, glutathione depletion, GPX4 inactivation, and increased lipid peroxidation [38]. The downregulation of antioxidant proteins (SLC7A11 and GPX4) and the upregulation of toxic derivatives (4-HNE and MDA) shown in the OVA group in this study demonstrates that ferroptosis signaling is involved in OVA-induced asthma pathogenesis. Lipid peroxides are decomposed into toxic derivatives such as 4-HNE and MDA [39], and MDA and 4-HNE are increased during ferroptosis [40].

Lipid peroxidation is a key factor that leads to asthma pathogenesis and exacerbation [9]. The serum MDA level has previously been found to be higher in asthmatics than in the healthy control group, and the MDA level is decreased by asthma treatment [41]. However, the levels of lipid peroxides have been found to be higher in asthmatics than in healthy controls, despite being well controlled by asthma treatment [42]. In our OVA group, ROS and MDA concentrations were markedly high compared with the CTL group. ER stress is known to be increased during ferroptosis [43,44]. ER stress markers (p-PERK, ATF4, and CHOP) have been found to be highly expressed in lung tissue obtained from an OVA-induced mouse model, and the PERK–ATF4–CHOP signaling has been found to be associated with airway inflammation [26,45]. The relationship between ER stress and ferroptosis indicates that crosstalk between ferroptosis and other types of cell death may be present. In our previous study, ER stress was related to inflammatory, apoptotic, ferroptotic, and pyroptotic signals in LPS-induced acute liver failure [35]. ER stress and oxidative stress frequently interact, and crosstalk between the two occurs [46].

ER stress and ferroptosis inhibitors could be proposed as new directions for asthma prevention and treatment. Few ferroptosis inhibitors have been identified so far. Lipophilic antioxidants, such as vitamin E, curcumin, epigallocatechin gallate, baicalein, and nordihydroguaiaretic acid, have been suggested as inhibitors of ferroptosis, as they compensate for GPX4 loss and prevent iron accumulation, GSH depletion, and lipid peroxidation [38]. Given the results of our study, we add FAGS and CK to the database of ferroptosis inhibitors. Here, the CK level among the ginsenosides distributed in FAGS increased the most compared with GS. CK has higher bioavailability and solubility than the parent ginsenosides [18]; furthermore, CK has been discovered in human tissues or blood after the oral consumption of PPD-type ginsenosides and red ginseng extract [19,20]. From our HPLC data and previous studies, the ginsenoside bioconversion mechanism can be estimated as follows: PPD type is converted to Rb1/or Rb2/or Rc→Rd→Rg3 or Rb1/or Rb2/or Rc→Rd→F2→CK [16,17]. The bioconversion mechanism of ginsenosides is that β-glycosidase and lactic acid produced by lactic acid bacteria convert high molecular weight ginsenosides containing several sugars into CK composed of one sugar. The increase in CK, known as the final intestinal metabolite of PPD-type ginsenosides in FAGS, shows that PPD-type ginsenosides in GS is bioconverted to CK by hydrolytic enzymes and metabolites of *L. brevis* and *L. plantarum*. Consuming fermented red ginseng extract has been shown to increase the plasma content of CK by over a factor of ten in comparison to ingesting unfermented red ginseng extract [47]. FAGS and CK can be said to be substances that can compensate for the low in vivo metabolism and stability, which are shortcomings of antioxidants used in clinical asthma treatment. In addition, CK has been shown to exhibit anti-inflammatory and anti-asthmatic effects by inhibiting airway resistance, IgE plasma levels, airway inflammation, and mucus secretion in asthma mouse models [23,48]. These characteristics fully suggest the potential of CK as a therapeutic agent for asthma. CK appears to be a substance that can modulate several signaling pathways detected in asthma pathogenesis.

In this study, the effect of CK alone on asthma relief was slightly more positive than that of FAGS. However, the CK concentration used in this study was almost 23 times higher than that in HFAGS. In many animal disease models and humans, CK concentrations higher than 30 μM have been used for treatment and have been effective and non-toxic in the models [18,23]. Therefore, in this study, 50 μM CK was used to determine the CK effect in asthma model. CK (89 μM and 112 μM) suppresses the arthritis scores in mice [49]. In addition to CK, many kinds of ginsenosides are also present in FAGS. Ginsenosides (F2, Rg3, Rg2, Rh1, and Ro) increased in FAGS may enhance the anti-asthmatic effect together with CK. Ginsenoside Rg3 has been described to ameliorate allergic airway inflammation and oxidative stress in mice [50]. However, the direct effects of ginsenoside F2, Rg2, Rh1, and Ro on asthma parameters have not yet been identified. Further studies are needed to determine the effects of other ginsenosides on asthma pathogenesis. Although the effects of all other ginsenosides have not been confirmed, CK in FAGS is considered sufficiently effective in asthma models. In a previous study, higher but quite low (4 μM) CK concentration than HFAGS showed an anti-inflammatory effect in an asthma model [48]. Further studies are needed to confirm the anti-asthmatic effect at the CK concentrations contained in LFAGS and HFAGS.

Many people tend to prefer nutraceuticals over drugs when looking after their health. Here, FAGS had high amounts of F2, Rg3, Rg2, Rh1, PPT, and Ro compared with GS. These components were increased by the fermentation and aging processes. GS is a representative medicinal vegetable with the aroma, taste, and active ingredients of ginseng, and it has a short cultivation period and similar ginsenoside contents compared with mature ginseng. FAGS is a unique GS that has been fermented and aged to increase its ginsenoside contents, which are low in GS. These properties of FAGS are factors that could make it a promising nutraceutical. Its efficacy and economic benefits hold high potential for its development as a nutraceutical. Based on our findings, we suggest that FAGS and CK may, respectively, be a nutraceutical that may alleviate asthma symptoms and potential asthma treatment, even though we still need more detailed studies.

## 5. Conclusions

In conclusion, FAGS and CK showed anti-asthmatic effects such as reduction in the following: airway hyperresponsiveness, IgE, inflammation, mucus production, collagen accumulation, Th2 IL, histamine, tryptase, calcium, ROS, MDA, cell death, macrophage infiltration, ER stress and ferroptosis signals, and iron accumulation in an OVA-exposed allergic mouse model. These results suggest that FAGS may be a potential dietary supplement to help treat oxidative and inflammatory diseases, including allergic asthma. The ferroptosis markers can be applied to asthma diagnostics, and CK can be a promising candidate for future asthma treatment.

## Data Availability

The study did not report any data.
